# Functional Segregation within the Muscles of Aquatic Propulsion in the Asiatic Water Monitor (*Varanus salvator*)

**DOI:** 10.3389/fphys.2016.00380

**Published:** 2016-09-08

**Authors:** Bruce A. Young, Jessica Dumais, Nicholas John, Brandon Lyons, Andrew Macduff, Matthew Most, Nathan A. Reiser, Peter J. Reiser

**Affiliations:** ^1^Department of Anatomy, Kirksville College of Osteopathic Medicine, A.T. Still University of Health SciencesKirksville, MO, USA; ^2^Department of Physical Therapy, University of Massachusetts LowellLowell, MA, USA; ^3^Department of Biosciences, College of Dentistry, Ohio State UniversityColumbus, OH, USA

**Keywords:** electromyography, whole muscle physiology, muscle histochemistry, work loops, myosin heavy chain, axial muscles, appendicular muscles

## Abstract

Water monitor lizards (*Varanus salvator*) swim using sinusoidal oscillations generated at the base of their long (50% of total body length) tail. In an effort to determine which level of the structural/organizational hierarchy of muscle is associated with functional segregation between the muscles of the tail base, an array of muscle features—myosin heavy chain profiles, enzymatic fiber types, twitch and tetanic force production, rates of fatigue, muscle compliance, and electrical activity patterns—were quantitated. The two examined axial muscles, longissimus, and iliocaudalis, were generally similar at the molecular, biochemical, and physiological levels, but differed at the biomechanics level and in their activation pattern. The appendicular muscle examined, caudofemoralis, differed from the axial muscles particularly at the molecular and physiological levels, and it exhibited a unique compliance profile and pattern of electrical activation. There were some apparent contradictions between the different structural/organizational levels examined. These contradictions, coupled with a unique myosin heavy chain profile, lead to the hypothesis that there are previously un-described molecular/biochemical specializations within varanid skeletal muscles.

## Introduction

Skeletal muscle is a dynamic tissue the functional properties of which are influenced, if not determined, at a variety of organizational levels. The diversity of myosin isoforms, as well as other molecular components within the single muscle fiber, can produce functional heterogeneity (Bottinelli, 2001). The packaging of the muscle fibers, both in terms of the geometric properties of the muscle (Azizi and Roberts, 2014) and the biomechanical properties of the muscle fibers and muscle-tendon complex (Fouré et al., 2012) will influence the timing and magnitude of force production. At a higher level of organization, the dynamics of the muscle-tendon complex will depend, in part, on the pattern of neural stimulation (e.g., Lacquaniti et al., 2012). While this is widely recognized, the majority of published studies focus on a single analytical technique applied to just one level of muscle organization.

One of the key evolutionary developments of craniates was an appendicular system (see Schilling, 2011), and increasingly studies are finding key differences between axial and appendicular muscles within the same individual. Differential regulation of myogenic pathways can generate molecular differences between appendicular and axial muscles (Rao et al., 1996; Spangenburg and Booth, 2003), and key developmental features of axial and appendicular muscles differ (Kablar et al., 1997; Neyt et al., 2000). Bagnall and McLean (2014) have described how the complexity of locomotor spinal circuits for axial control could serve as a “source” for the early neural pattern for tetrapedal (appendicular) locomotion.

The monitor lizards (*Varanus*) have diversified, both biogeographically and ecologically (Ast, 2001), in ways that make them attractive model organisms for studies of muscle function. Although the basic body plan of the varanids is conserved (despite the largest size range of any terrestrial vertebrate radiation, Pianka et al., 2004), the group shows distinct specializations in limb allometrics and tail shape that are associated with a shifting emphasis from appendicular to axial locomotion (Christian and Garland, 1996; Burnell et al., 2012a). These locomotor specializations are superimposed onto a suite of physiological specializations for increased aerobic capacity (see Frappell et al., 2002) and endurance. An earlier study (Young et al., 1990) documented a range of functional characteristics within the shoulder muscles of the savannah monitor (*Varanus exanthematicus*), a species that relies on appendicular locomotion. The present study concentrates on the water monitor (*Varanus salvator*), a species that swims using axial undulations, primarily of the tail base (Young et al., 2008; Burnell et al., 2012a). As a comparison to the earlier study (Young et al., 1990), the present study applied a variety of analytical techniques to two axial muscles of the tail base, as well as one appendicular muscle (caudofemoralis) which is a major retractor of the femur but has been postulated to laterally displace the tail base (Gatesy, 1990, 1997; Caldwell and Sasso, 2004). By comparing these three muscles, two axial and one appendicular, from within these physiologically specialized lizards, we hope to explore patterns of functional specialization both within and between these skeletal muscles.

## Materials and methods

### Live animals

Eight specimens of the Asian water monitor (*V. salvator*) were obtained commercially. To minimize scaling influences, all of the specimens had a similar total body length (85–120 cm). The animals were maintained individually in terraria with 12:12 light cycle, water *ad libitum*, a temperature range of 29–34°C, and a diet of pre-killed rodents. Maintenance and use of these animals followed all applicable guidelines for vertebrate animals, and were approved by the Institutional Animal Care and Use Committee (Registration # 14-R-0081).

### Kinematics and electromyography

**Figure 1:**
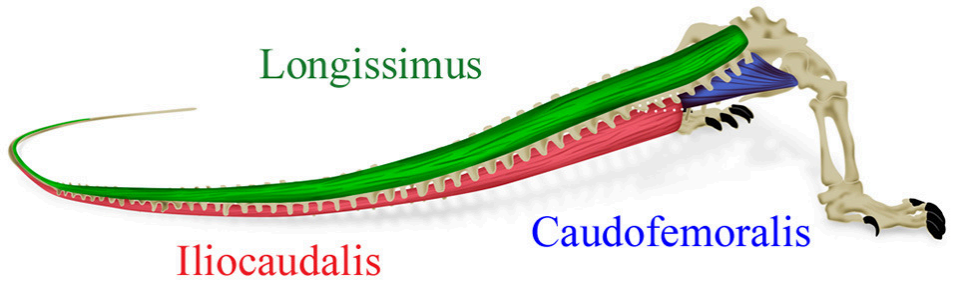
**Simplified diagram of the three studied muscles**. The proximal base of the iliocaudalis (in red) has been removed to better illustrate the deeper caudofemoralis (in blue).

Muscle activity patterns were quantified from four specimens of *V. salvator*. Earlier analysis had documented that there was little undulatory displacement of the trunk during swimming in this species (Burnell et al., 2012a), so the present analysis focused exclusively on muscles of the tail base. With the specimen lightly anesthetized with Isoflurane, sterile hypodermic needles were used to implant bipolar electromyographic (EMG) leads (fabricated from 0.05 mm diameter stainless steel wire with Nylon insulation; California Fine Wire, Grover City, CA) into the caudofemoralis, the longissimus (an epaxial muscle) and the iliocaudalis (a hypaxial muscle). The latter two muscles were implanted within the proximal 5% of the tail length (Figure 1).

When the specimen was fully recovered from anesthesia, it was placed into a 148 × 63 × 45 cm deep re-circulating flow tank (based on Vogel and Labarbera, 1978) which was filled with water maintained at 25–26°C. A TroubleShooter 1000S (Xcitex Inc., Woburn, MA) high-speed digital video camera was mounted over the flow tank and used to record the swimming sequences at either 125 or 250 fps. The resulting sequences were screened to eliminate those in which (1) the animal completed less than two complete tail oscillations; (2) the animal's trajectory deviated by more than 10° (measured as a line through the axis of the skull to the midline of the pelvis of each frame); or (3) the swimming velocity of the animal (see below) changed significantly during the sequence. A minimum of five swimming sequences from each specimen were used for analysis. MaxTRAQ software (Innovision Systems Inc., Columbiaville, MI) was used to control the camera and isolate sequence frames which were exported to ImageJ (NIH) for digitization. Using Excel and SSPS software packages, the swimming velocity of the lizard (defined as change in position of the snout tip over time) and the amplitude of each undulatory peak in the tail were calculated.

The EMG leads were bandpass filtered (10 Hz–1 kHz) and preamplified (QP511, GRASS Technologies, Natus Neurology Inc., Warwick, RI) prior to A/D conversion and recording (at 10 kHz) using MIDAS (Xcitex, Inc.). A pulsed LED strobe/voltage spike, generated by a S88 stimulator (GRASS Technologies) synchronized the EMG and kinematic data sets. Using the MIDAS and EXCEL software packages, the onset and offset times of the muscles were quantified, as was the area under the rectified muscle activation signal.

### Whole muscle physiology

Four specimens were anesthetized with Isoflurane, and the base of the tail was surgically exposed for *in situ* analysis. At the same transverse plane of the tail, and always within the proximal 5% of the tail, the attachments of the caudofemoralis, longissimus, and iliocaudalis were surgically isolated from the caudal vertebrae. The proximal attachment of each muscle was clamped (via the bony pelvis or knee) and the surgically-isolated end was attached to an FT10 force transducer (GRASS Technologies) via silk suture. The muscle was stimulated using an S88 stimulator (GRASS Technologies) and a bipolar stimulating electrode. The force transducer was connected to a P122 strain gage amplifier (GRASS Technologies), the signal from which was recorded (at 10 kHz) using the MIDAS data acquisition software and hardware. A synchronized pulse from the S88 stimulator was recorded with the output of the force transducer, providing a 0.1 ms temporal resolution to the muscle physiology data.

The same sequence was followed for each muscle; (1) an initial series of at least 12 twitch contractions, with a minimum of 1 s between contractions; (2) two tetanic contractions recorded with at least 2 min between the contractions; and (3) a 2 min fatigue test during which the muscle was stimulated at a rate (1 tps, 40 Hz, 330 ms) that produced repeated summation but not tetanus. Upon completion of this series, a transverse section through the muscle was excised, digitally photographed, and the cross-sectional area quantified using ImageJ (NIH). The FT10 force transducer was then calibrated by vertically suspending reference masses.

Using the MIDAS software and EXCEL, the following were quantified: twitch contraction time, twitch force production (standardized by muscle cross-sectional area), tetanic force production (standardized by muscle cross-sectional area), ratio of twitch/tetanic force, and rate of muscle fatigue.

### Muscle compliance

Three specimens were anesthetized with Isoflurane, and the caudofemoralis, longissimus, and iliocaudalis muscles were surgically isolated, as described above. With the proximal attachment of the muscle clamped, the surgically-isolated end of the muscle was connected to an FT10 force transducer (GRASS Technologies) using a small stainless steel clamp. The force transducer was mounted onto a linear actuator (PPS2-17, Picard Industries). This actuator was chosen because its cycling frequency (1 Hz) and throw distance (1.8 cm) were similar to the oscillating frequency and amplitude (1.2 Hz and 4.2 cm, respectively) of the tail base movements measured from the kinematic records of swimming *V. salvator*.

The actuator/force transducer array was positioned so that the midpoint of the throw corresponded to the muscle length when the tail was in the neutral (sagittal) plane. The force transducer was connected to a P122 strain gage amplifier (GRASS Technologies), the signal from which was recorded (at 10 kHz) using the MIDAS data acquisition software and hardware. Constant voltage to the actuator cycled the muscle through a series of passive elongation and shortening; little variation was observed in the resulting passive force curves. After a minimum of 20 cycles were recorded, twitch stimuli were applied to the muscle (as described above) while the muscle was being cycled by the actuator. The timing of the twitch stimuli varied relative to the cycle of the muscle/actuator; when a series of twitch stimuli curves (each taken at slightly different points of the muscle/actuator cycle) are combined it creates an active force curve. The two ends of the passive and active force curves both represent the starting length; the X-axis of the curve could be changed to length (instead of time) which would create a work loop. Note that this work loop would differ from the traditional muscle work loop in being restricted to just the central portion of the muscle's possible operating length.

### Muscle enzymatic histochemistry

Upon completion of the whole muscle physiology procedures (see above), transverse sections were excised from the caudofemoralis, longissimus, and iliocaudalis on the contralateral (unstimulated side) of the lizard. The blocks were flash frozen using an isopentane bath suspended in liquid nitrogen. Fourteen micron sections were cut (at −20°C) using an OTF Cryostat (Bright Instruments, Cambridgeshire, England). To characterize the muscles as either slow-oxidative (SO), fast-glycolytic (FG), or fast-oxidative glycolytic (FOG) we assayed ATPase, α-glycerophosphate dehydrogenase (α-GPDH), and succinic dehydrogenase (SDH) activities using established techniques (Gleeson et al., 1980).

### Myosin heavy chain isoform composition

Muscle tissue was extracted from the same muscles and specimens used for muscle enzymatic histochemistry (above) and from the contralateral caudofemoralis, iliocostalis, and longisimmus from two other specimens. From all five specimens, the heart was excised as part of the euthanasia protocol, and tissue samples were taken from both the ventricle and the atria. From one of the specimens, samples were also excised from transversospinalis, biceps brachii, and triceps brachii. Muscle samples were frozen and stored (at either −20 or −80°C); samples from two of the animals were placed in glycerinating solution (Bergrin et al., 2006) to render the fibers in a hyperpermeable (skinned) state and stored at −20°C. This allowed the subsequent isolation, by dissection, of single fibers that contained essentially only myofibrillar proteins, as the soluble proteins diffused from the fibers. The frozen and glycerinated samples were shipped overnight to Ohio State University within 2 days following euthanasia.

The frozen (not glycerinated) samples were thawed and prepared as described by Blough et al. (1996). Briefly, 30 μl of sample buffer were added per mg of tissue and the samples were homogenized for 5–10 s. The samples were heated (65°C for 2 min), chilled on ice for 5 min, centrifuged, and the supernatant was diluted 1:10 with the same sample buffer before gel loading, unless indicated otherwise. Sample volume was 3 μl. Except for one gel run (described below), all of the separating gels consisted of 7% acrylamide (50:1 acrylamide:bis cross-linking ratio) and 30% glycerol and were run at 8°C for 1.5 h at constant 275 V, then for another 25–27 h at constant 300 V in SE600 Hoefer gel units. This was the gel format that yielded the greatest number of MHC bands, in several reptilian species, with high resolution among multiple tested formats, as recently reported (Reiser and Bicer, 2014). All of the gels were silver-stained (Blough et al., 1996). The amount of each MHC isoform in each sample of caudofemoralis, iliocaudalis, and longissimus was determined by scanning densitometry using a GS300 scanning densitometer (Hoefer Scientific; San Francisco, CA), as described in Bicer and Reiser (2004). The relative amount (as a percentage of total MHC) of each MHC isoform in each sample was calculated. Differences in the mean relative amount of each isoform were compared among the caudofemoralis, iliocaudalis and longissimus, using analysis of variance (SYSTAT) and the *t*-test for *post-hoc* determination of statistical significance.

### Myosin heavy chain isoform identification

An immunoblot with an antibody that recognizes all sarcomeric MHC isoforms (MF 20, diluted 1:50, obtained from the Developmental Studies Hybridoma Bank at the University of Iowa) was run to verify that all of the scanned bands were MHC isoforms. Another antibody (1170-S, clone F88-12F8, diluted 1:50; BioCytex, Marseille, France) was used to test for the expression of cardiac MHC-α. All of the steps were conducted at room temperature. Proteins were transferred to nitrocellulose membranes, using a mini-V BRL unit (Life Technologies, Gaithersburg, MD). Transfers were run at constant 100 volts for 2 h. The transfer buffer composition was 25 mM Tris, 192 mM glycine, 20% (v/v) methanol, 0.08% (v/v) beta-mercaptoethanol and 0.1% (w/v) SDS. Blocking of the membranes was performed with 1% bovine serum albumin (BSA) in Tris-buffered saline with 0.05% (v/v) Tween 20 (TBST) for 1 h. The membranes were reacted with the primary antibodies in TBST with 0.3% BSA for 1 h and were washed three times, 5 min each, in TBST. The membranes were incubated, for 1 h, with an alkaline phosphatase-conjugated secondary anti-mouse antibody (S3721, Promega, Madison, WI; 3 μl in 20 ml of TBST with 1% BSA,) and were washed three times, 5 min each, in TBST. Color development was performed with 5-bromo-4-chloro-3-indolyl phosphate and nitro blue tetrazolium dye as substrates (Promega).

Differences in separating gel composition can alter the electrophoretic mobility of some MHC isoforms relative to others and can yield separation of some isoforms that are not separated from each other on other gel formats (e.g., Reiser and Bicer, 2014). Therefore, some samples were also run on separating gels that consisted of 9% acrylamide, 200:1 crosslinking, and 12% glycerol, to test whether any monitor MHC isoforms that co-migrated on one format could be separated from each other on a different format. If a difference between formats is observed, then this would indicate that the isoforms are, in fact, different. Gels with this format were run at 18°C and at constant current, at 10 mA/gel for the first 2.5 h and 20 mA/gel for 25.5 additional hours. The stacking gels used with this separating gel format consisted of 4% acrylamide, with an acrylamide:bis cross-linking ratio of 50:1, 0.125 M Tris, pH 6.8 and 0.1% (w/v) SDS.

One gel, with the first format described above (i.e., 7% acrylamide (50:1 acrylamide:bis cross-linking ratio) and 30% glycerol) was loaded with samples of atrium, ventricle and caudofemoralis. The caudofemoralis sample was observed, on previous gels, to contain all five MHC bands in monitor skeletal muscles. The gel was stained with Coomassie Blue and both atrial bands, the single ventricular band and bands 1, 2, and 5 (**Figure 8**) from the caudofemoralis sample were excised and submitted for identification by mass spectrometry at the Campus Chemical Instrument Center at Ohio State University. Bands 3 and 4 were not analyzed because of their low abundance and greater sample loads created contaminating trailing edges from the bands that migrated immediately in front of these two bands. The bands were subjected to Orbitrap Fusion nanoLC-MS/MS. Collision-induced fragmentation, coupled with electron-transfer dissociation, was used to generate peptides. The fragment mass tolerance was set at 0.8 Dalton. The results were blasted against the SwissProt database for protein identification.

Skinned fibers were isolated from strips of biceps brachii, caudofemoralis, longissimus, and iliocaudalis (23 or 24 fibers from each muscle) that had been stored at −20°C in glycerinating solution. Fibers were isolated by dissection in a Petri dish containing relaxing solution (composition as described in Bergrin et al., 2006). Two types of fibers, based upon appearance when viewed with a dissecting microscope (either small diameter and white (semi-opaque) or large diameter and clear) were easily discerned and this was recorded for each isolated fiber. The fiber diameter was estimated by inspection and fiber length was measured with a ruler in the dissecting dish. Fiber volume was calculated from the diameter estimate (assuming a circular cross-section) and length measurement. Each fiber was transferred to an individual microcentrifuge tube and 2.0 μl of sample buffer per ηl of fiber volume were added to each single fiber tube. The tubes were heated for 5 min at 65°C, then chilled immediately on ice for 5 min. The fibers were stored at −40°C until loading onto a gel. Three microliters of each single fiber sample were loaded onto gels which were silver-stained. All 24 fibers from a given muscle were loaded onto two gels and a sample of a homogenate of the same muscle, which was known to contain all five MHC isoforms in monitor skeletal muscles, was loaded in the center lane of each gel, as a standard to identify the MHC isoform(s) in each single fiber. The pattern of MHC isoform content of each fiber was determined by inspection of the gels.

## Results

### Electromyographic activity pattern

**Figure 2:**
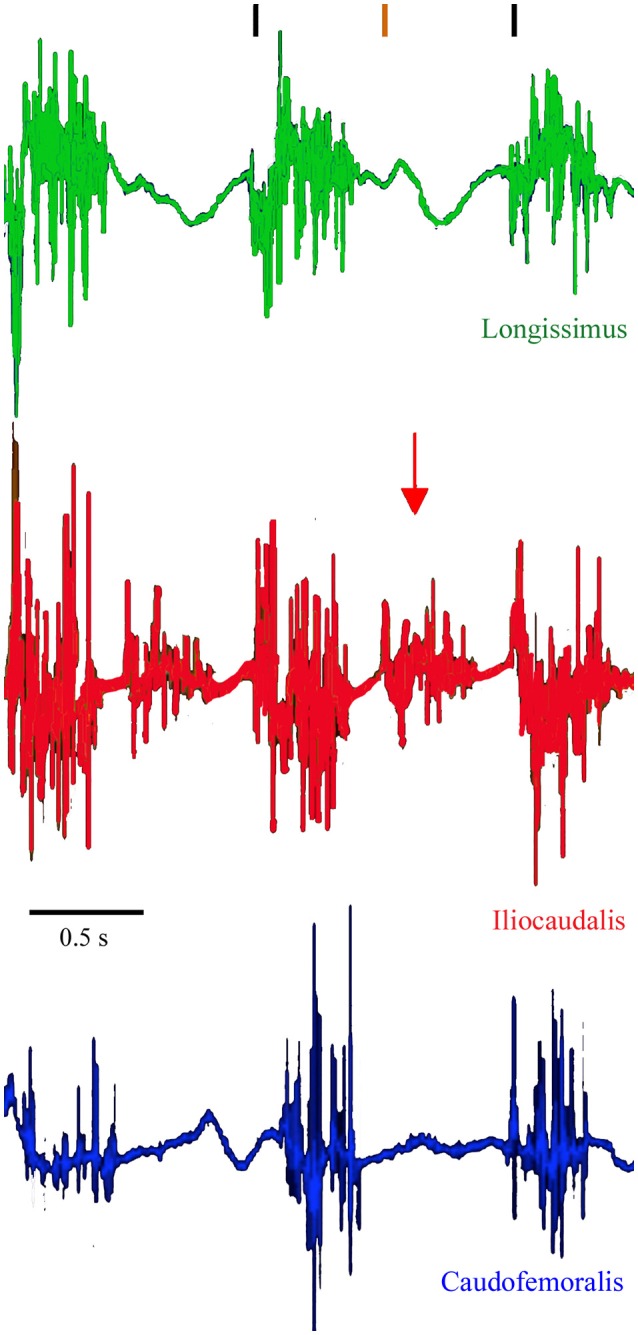
**Raw EMG traces from a free swimming *Varanus salvator***. The onset of tail displacement is indicated by the black vertical lines (top), the peak lateral displacement (and reversal of direction) is indicated by the orange vertical line. Green trace is longissimus, red trace is iliocaudalis, and the blue trace is caudofemoralis. Note the second burst of activity in iliocaudalis associated with the change in direction of the tail sweep (red vertical arrow).

**Figure 3:**
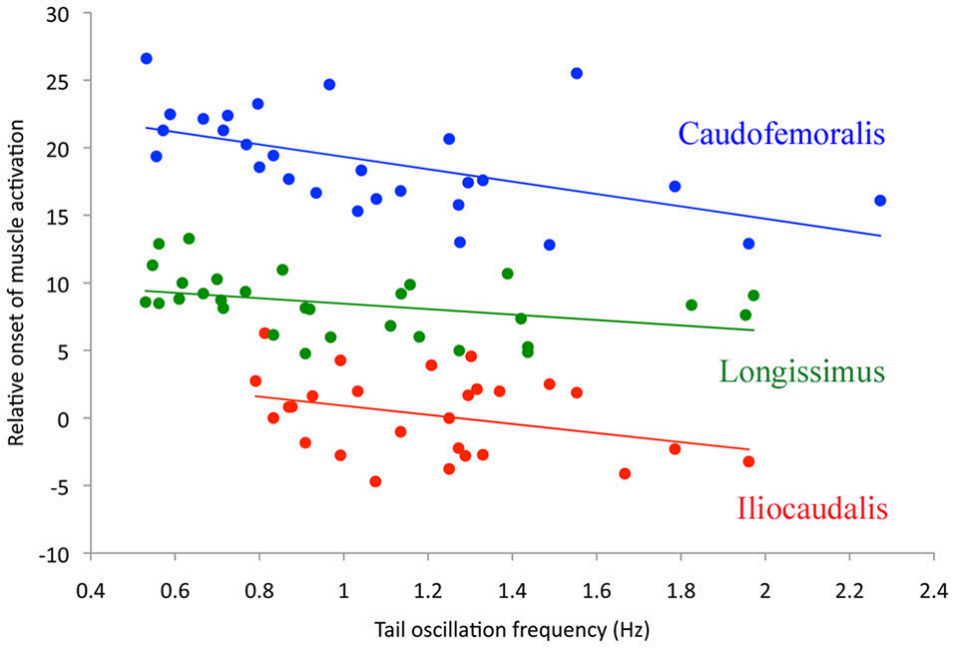
**Timing of the onset of muscle activation (as a percent of total tail cycle time, Y-axis) as a function of swimming speed (represented by tail oscillation frequency, X-axis)**. There is a clear sequence of muscle activation starting with iliocaudalis (red), then longissimus (green), and finally caudofemoralis (blue). This pattern held over the 3x range of tail oscillation frequencies observed.

**Figure 4:**
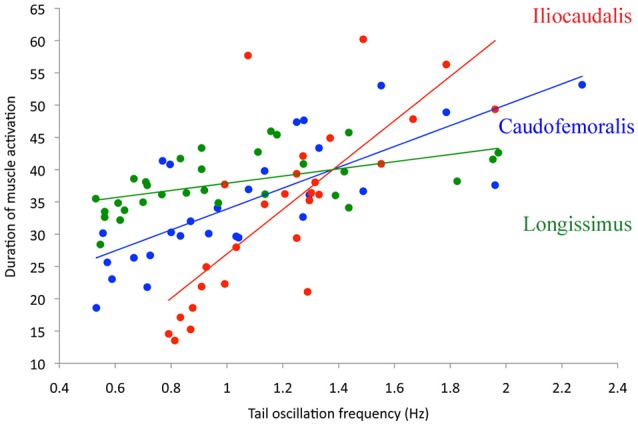
**Duration of muscle activation (as a percent of total tail cycle time, Y-axis) as a function of swimming speed (represented by tail oscillation frequency, X-axis)**. The iliocaudalis (red) and caudofemoralis (blue) are active for more of the tail cycle at higher swimming speeds, while the longissimus (green) is statistically constant.

The EMG patterns of activation of the three muscles differed from each other. The iliocaudalis EMG pattern differed from the pattern of the other two muscles in showing two bursts, one associated with the onset of tail oscillation, and the second when the tail reversed direction halfway through the oscillation cycle (Figure 2). The iliocaudalis fired shortly before tail movement began, followed (after approximately 10% of the oscillation cycle duration) by the longissimus, then (after approximately 20% of the oscillation cycle duration) the caudofemoralis (Figure 3). The differences among these onsets were significant (ANOVA, *F* = 244.9, 2 df, *p* < 0.0001) as was the decrease in relative onset time within the iliocaudalis and caudofemoralis with increasing tail oscillation frequency (*b* = −4.13, *F* = 22.42, *df* = 25, *p* < 0.0001 and *b* = −4.58, *F* = 10.87, *df* = 26, *p* = 0.0028, respectively). The relative onset time of the longissimus did not change with increasing oscillation frequency (*b* = −1.715, *F* = 3.24, *df* = 28, *p* = 0.075). The duration of electrical activity in the iliocaudalis and caudofemoralis increased with increasing tail oscillation frequency (*b* = 34.38, *F* = 37.26, *df* = 25, *p* < 0.0001 and *b* = 16.2, *F* = 36.29, *df* = 26, *p* < 0.0001, respectively; Figure 3); the duration of the longissimus did not significantly change with increasing tail oscillation frequency (Figure 4).

### Whole muscle physiology

**Figure 5:**
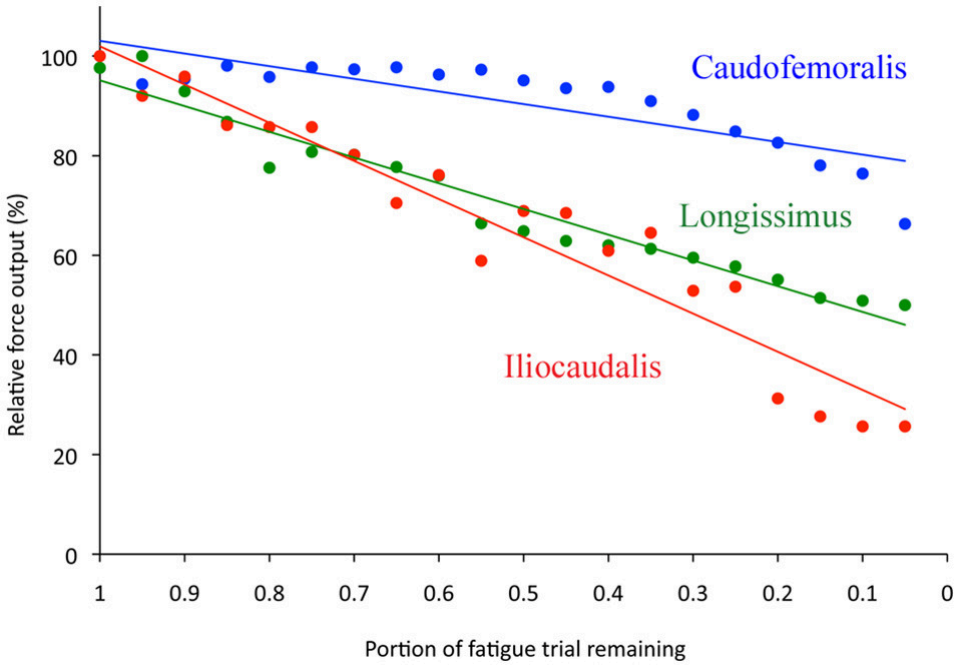
**Results of the 2-min fatigue trials**. The longissimus (green) and iliocaudalis (red) show a similar fatigue profile; the caudofemoralis (blue) is more resistant to fatigue.

The mean cross-sectional area of the iliocaudalis (49.6 mm^2^) was roughly 65% that of the caudofemoralis and of the longissimus (Table 1). The twitch contraction time of the caudofemoralis (mean = 154 ms) was significantly (ANOVA, *F* = 12.5, 2 df, *p* < 0.0001) longer than that of the longissimus or iliocaudalis (which were not significantly different from each other, Table 1). The twitch forces of the three muscles (when normalized with cross-sectional area) were all significantly different (ANOVA, *F* = 1051, *df* = 2, *p* < 0.0001); the force produced by caudofemoralis being roughly double that of the longissimus (Table 1). The tetanic forces yielded a similar pattern; the three muscles were different (ANOVA, *F* = 87.6, *df* = 2, *p* < 0.0001) and caudofemoralis had nearly twice the force output of the longissimus (Table 1). The tetanic forces were approximately 5x those of the twitch forces (Table 1); the ratio being slightly (but significantly; ANOVA, *F* = 161.5, *df* = 2, *p* < 0.0001) higher at 5.7 in the longissimus. The caudofemoralis exhibited the lowest rate of fatigue (Figure 5) retaining >80% of twitch force at the termination of the 2 min trial. The fatigue rate (calculated as linear regression of relative force output per unit interval of the fatigue test) for the iliocaudalis was nearly 3x that of the caudofemoralis; the fatigue rate for the longissimus was halfway between those of the other two muscles (Figure 5, Table 1).

**Table 1:** **Summary of the whole muscle physiology and enzymatic histochemistry of the three muscles examined**.

	**Caudofemoralis appendicular**	**Iliocaudalis hypaxial**	**Longissimus epaxial**
Cross-sectional area (mm^2^)	80.2 (2.2)	49.6 (1.9)	78.3 (2.5)
Twitch contraction time (ms)	154 (10)	115 (17)	110 (16)
Twitch force (N/mm^2^)	2.0 (0.20)	1.4 (0.25)	0.9 (0.09)
Tetanic force (N/mm^2^)	9.83 (0.99)	6.8 (0.59)	5.13 (0.42)
Tetanic force/twitch force	4.92	4.86	5.7
Fatigue rate	−1.27	−3.83	−2.58
Histochemical Profile			
SO%	72	65	61
FG%	11	10	30
FO%	17	25	9

Physiological values are expressed as mean (s.e.); fatigue rate was calculated as the linear regression of relative force output per unit interval of the fatigue test.

### Muscle compliance

**Figure 6:**
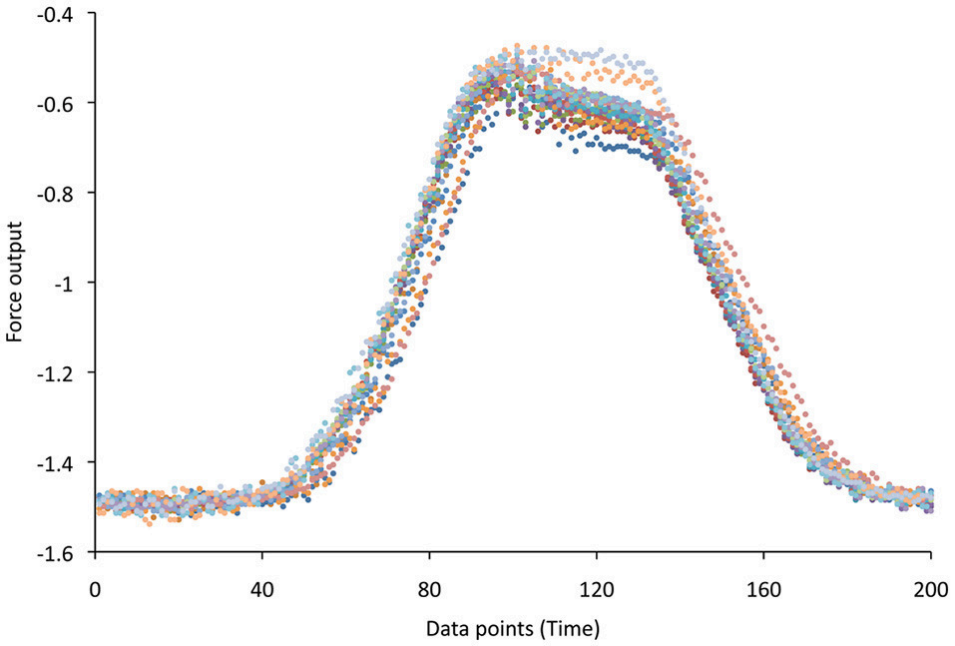
**Raw data traces from a force transducer (Y axis) coupled to a linear actuator and clamped to iliocaudalis**. By applying a constant voltage to the linear actuator it was possible to induce a regular pattern of passive elongation and shortening in the muscle. The different colors represent different elongation/shortening cycles; each point is a single measurement.

**Figure 7:**
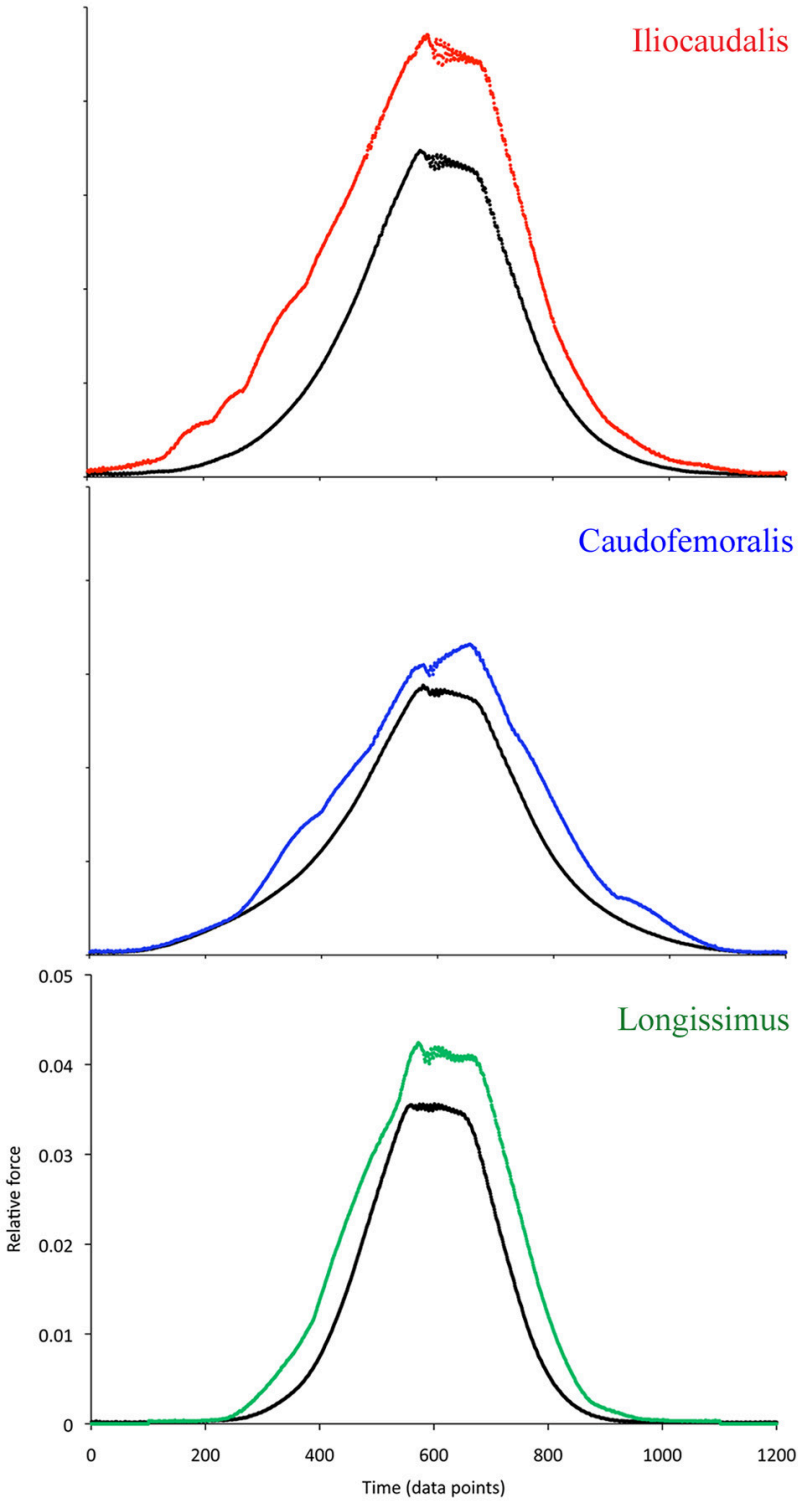
**Muscle compliance curves showing the passive curve (black) and the active curves for the iliocaudalis (red), caudofemoralis (blue), and longissimus (green)**. The X-axes (time) and Y-axes (force) are constant for all three sets of curves. Note the difference in the shapes and magnitudes of both the passive and active curves.

The cyclic movements of the actuator were capable of producing repetitive length changes in the muscles (Figure 6). When the relative force of the muscle was adjusted for cross-sectional area and plotted against time (length), there were distinct differences among the three muscles (Figure 7). The passive force curve (the black line in Figure 7) for caudofemoralis had the greatest response at low length changes, the lowest peak force, and the lowest slope. The passive curves for the two axial muscles were similar, although the iliocaudalis had more response at low length changes than did the longissimus. The active force curve (color coded in Figure 7) was proportionately similar in caudofemoralis and longissimus; the iliocaudalis produced greater active force (relative to passive force) and showed a more asymmetric profile with greater force production during active lengthening than shortening.

### Enzymatic fiber type profiles

The three muscles all had a predominance of SO fibers (Table 1). Among the fast-twitch fibers observed, those of the caudofemoralis and iliocaudalis were mainly oxidative, while those of the longissimus were mainly glycolytic. In none of the muscle sections examined was the distribution of the enzymatic fiber types suggestive of compartmentalization.

### Muscle and single fiber MHC isoform expression

**Figure 8:**
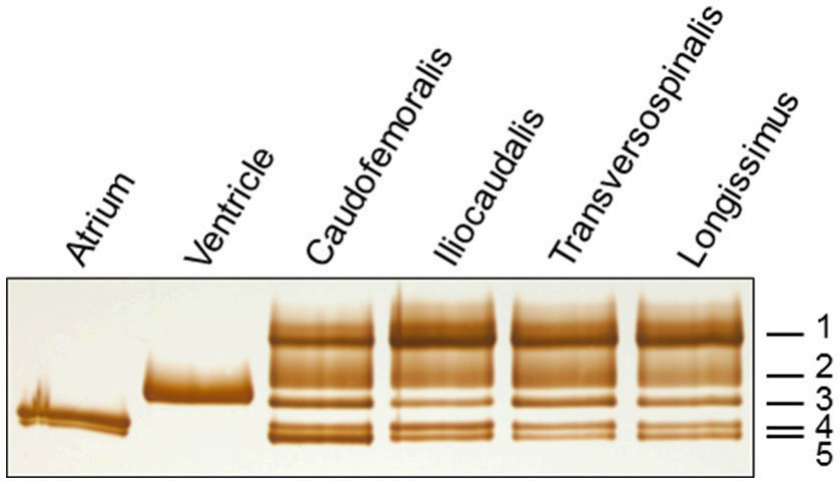
**The myosin heavy chain region of a silver-stained gel loaded with homogenates of cardiac and skeletal muscles**. The MHC band numbers refer to the MHC isoforms in the skeletal muscle samples.

The electrophoretic separation of MHC isoforms in the cardiac atrium and ventricle, caudofemoralis, iliocaudalis, transversospinalis, and longissimus is shown in Figure 8. Five MHC isoforms were consistently detected in the skeletal muscles that were examined from each animal. Each band was recognized by MF 20 antibody (not shown) and was, therefore, identified as an MHC isoform. The five bands observed in skeletal muscle samples are labeled as “1” (slowest migrating) through “5” (fastest migrating), as the identity was determined for some, but not all of the bands (see mass spectrometry results, below). The cardiac MHC bands are referred to as either “atrial” (two isoforms) or “ventricular” (one isoform). The relative amount of each band in the caudofemoralis, iliocaudalis and longissimus from seven *V. salvator* was determined (Table 2). Band 1 was expressed as the predominant MHC isoform in all muscles, although the relative amount was significantly greater in longissimus than in caudofemoralis. There appeared to be two bands, on some gels, in the region that is labeled as “Band 1” but this was not consistently observed. Therefore, it is regarded as one MHC band in this report. Band 2 was the second-most abundant band in each muscle (~20–30% of total MHC). The amount of Band 2 was significantly greater in iliocaudalis, compared to longissimus. Significant differences existed in the relative amounts of band 3 and band 5 (both were greater in the caudofemoralis than in either the iliocaudalis or longissimus). The relative amount of band 4 did not differ significantly among these muscles.

**Table 2:** **Relative amount (percent of total, given as mean ± s.e.) of each MHC isoform in homogenates of the three muscles**.

	**Caudofemoralis**	**Iliocaudalis**	**Longissimus**
% MHC 1	44.4 ± 5.2^*^	55.5 ± 5.0	68.4 ± 5.0^*^
% MHC 2	24.0 ± 3.3	30.9 ± 2.5^†^	20.4 ± 2.7^†^
% MHC 3	11.2 ± 1.8^Δ°^	2.3 ± 0.8^Δ^	4.5 ± 1.7°
% MHC 4	4.6 ± 1.6	3.3 ± 1.4	3.4 ± 1.6
% MHC 5	15.8 ± 1.9^+§^	7.9 ± 2.9^§^	3.3 ± 1.1^+^

N = 7 for each muscle. Values with the same symbol differ significantly (P < 0.05) from each other.

**Figure 9:**
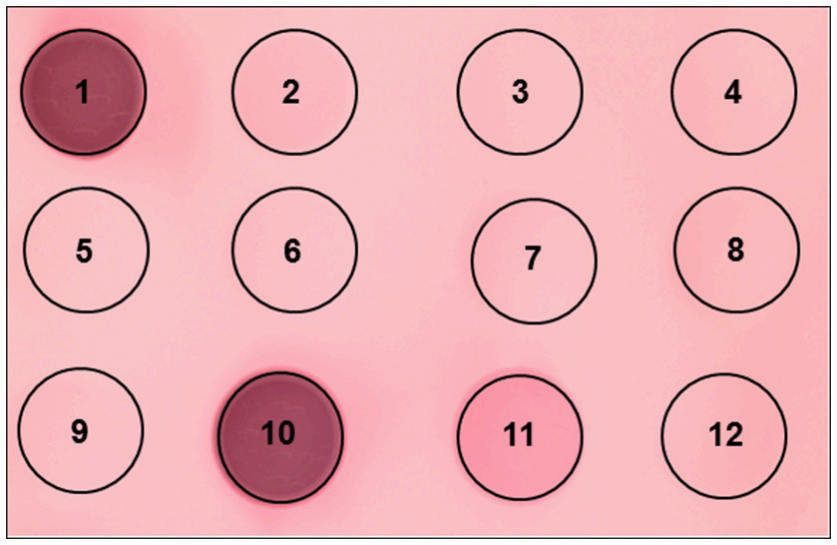
**Dot blot to determine whether the cardiac MHC-α isoform was present in the probed samples (all monitor, unless indicated)**. Top row (left to right): rat atrium (1), atrium (2), ventricle (3) and triceps (4). Middle row: longissimus (5), iliocaudalis (6), biceps (7) and caudofemoralis (8). Bottom row: transversospinalis (9), rat atrium (10), atrium (11, undiluted) and ventricle (12, undiluted).

**Figure 10:**
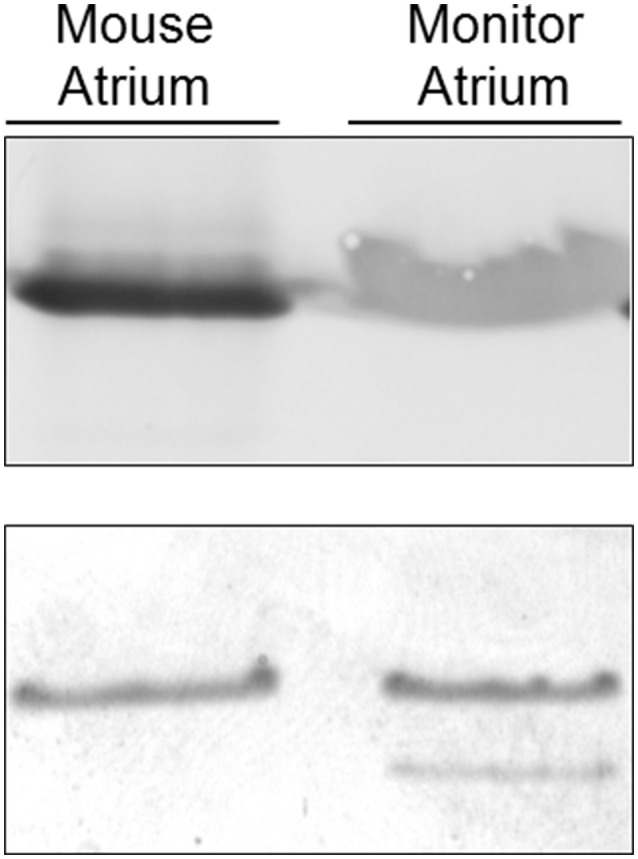
**Top:** immunoblot with a cardiac MHC-α antibody. Samples were undiluted (see Methods) mouse and monitor atria. **Bottom:** myosin heavy chain region of a silver-stained gel loaded with 1:10 diluted (see Methods) samples of mouse and monitor atria.

Two MHC bands were observed in the atrial samples (Figure 8). The slower migrating atrial band predominated in amount and this band co-migrated, on separating gels that contained 7% acrylamide and 30% glycerol, with band 4 in the examined skeletal muscles, while the minor atrial band co-migrated with skeletal muscle band 5. MHC-α is abundantly expressed in cardiac atria of all examined mammalian species, but is not typically expressed in mammalian limb muscle, although it is expressed in some craniofacial muscles in several mammalian species (e.g., Bredman et al., 1991, 1992; Pedrosa-Domellöf et al., 1992; Rahnert et al., 2010; Osterlund et al., 2012). A dot blot was used to test whether MHC-α is expressed in monitor atrium, ventricle or in triceps brachii, longissimus, iliocaudalis, biceps brachii, caudofemoralis, transversospinalis, since all of these muscles have two bands with similar electrophoretic mobility (Figure 9). Rat atrium was included on the dot blot as a positive control and the results indicated that MHC-α is, in fact, expressed in monitor atrium and not in any of the other muscles, including monitor ventricle. The affinity of the antibody for monitor MHC-α is apparently less than for rat MHC-α, but the former was positively identified with the undiluted monitor atrial sample (dot 11 in Figure 9). We then attempted to differentiate the mobility of the two atrial MHC bands and skeletal muscle bands 4 and 5, using gels with 9% acrylamide and 12% glycerol (see Methods). The two MHC isoforms in the atrial samples separated much farther from each other on this format, whereas the separation of bands 4 and 5 in the skeletal muscle samples was the same as on the other gel format (7% acrylamide and 30% glycerol). The predominant atrial band migrated much slower than either band 4 or 5 in skeletal muscles, whereas the minor atrial band co-migrated with band 5 in skeletal muscles. Another 9% gel was run with samples of mouse atrium (known to express almost exclusively MHC-α; e.g., Reiser and Kline, 1998) and monitor caudofemoralis. Both of these samples were loaded on the right and left half of one gel. One half was stained to visualize the alignment of mouse MHC-α and the two monitor atrial MHC bands and the other half was used for a western blot. The blot results revealed that only one monitor atrial band was recognized by the antibody and this band co-migrated with mouse MHC-α (Figure 10). The stained half of the gel indicated that mouse MHC-α co-migrated with the predominant band in monitor atria. The gel and immunoblot results collectively indicate that (1) the slower migrating, predominant atrial band in monitor atrium is homologous to MHC-α and (2) this isoform is not expressed in monitor ventricle or any of the examined skeletal muscles.

**Figure 11:**
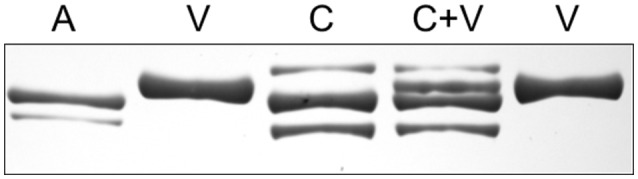
**The myosin heavy chain region of a silver-stained gel loaded with homogenates of the atrium (A), ventricle (V), and caudofemoralis (C)**. Three bands are visible in the caudofemoralis sample on this gel format (7% acrylamide and 5% glycerol). A fourth band is evident when the caudofemoralis and ventricle are loaded in the same lane (C+V), demonstrating that the caudofemoralis does not express the ventricular isoform.

**Figure 12:**
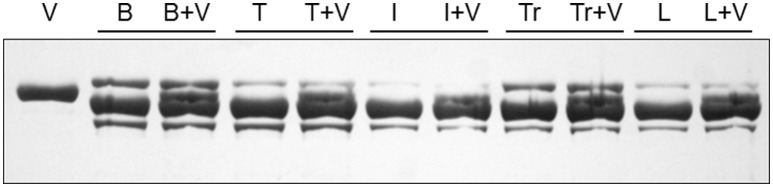
**The myosin heavy chain region of a silver-stained gel (7% acrylamide and 5% glycerol) loaded with homogenates of the ventricle (V), biceps (B), triceps brachii (T), Iliocaudalis (I), transversospinalis (Tr), and longissimus (L)**. Each sample was loaded alone in one lane and with a sample of the ventricle in the adjacent lane. Three bands are visible in each lane loaded only with a limb/trunk muscle and a fourth band was visible in each combined lane, illustrating that the ventricular MHC isoform is not expressed in the examined limb/trunk muscles.

All of the ventricular samples contained only one MHC isoform and its migration was distinct from that of the two atrial isoforms. The ventricular isoform appears to not be expressed in any of the examined skeletal muscles. To illustrate the unique mobility of the ventricular MHC band, additional gels, with 7% acrylamide and 5% glycerol, were run with limb or trunk skeletal muscles which were loaded either alone, in a given lane, or along with a sample of the ventricle in an adjacent lane (Figures 11, 12). The results show that the ventricular MHC band has a lower mobility than band 3 in skeletal muscle and is a distinct isoform from all of the isoforms in the examined limb/trunk muscles.

The MHC isoform compositions of single fibers from the biceps, longissimus and caudofemoralis of one monitor and the iliocaudalis from another monitor were determined using gel electrophoresis to evaluate the complexity in MHC isoform expression at the cellular level (Table 3). The biceps was included in this part of the study because homogenates contained virtually only bands 1 and 2 and an objective was determine if these are either expressed independently or co-expressed in individual fibers. Eighteen biceps fibers expressed exclusively MHC band 1, three expressed exclusively band 2 and two co-expressed bands 1 and 2. None of the examined biceps fibers expressed bands 3, 4, or 5. Half of the caudofemoralis fibers expressed a single MHC isoform—band 1, band 3 or band 5; the remaining fibers co-expressed MHC bands 1 and 2, 2 and 4, or 3 and 5. The distribution of MHC isoforms in single fibers of the longissimus was similar to that of the caudofemoralis, with the addition of two fibers that expressed exclusively MHC band 2 and none of the sampled longissimus fibers expressed only band 5. The predominant pattern in the examined iliocaudalis fibers was co-expression of bands 1 and 2 and, unlike the other muscles, none of the fibers expressed only band 1. Therefore, distinct patterns of MHC isoform expression were observed among the four muscles that were examined. Generally, the fibers in each muscle that had a small diameter and a white, semi-opaque appearance expressed bands 3 and 5, whereas the fibers with large diameters and a clear appearance expressed bands 1, 2, and 4.

**Table 3:** **MHC isoform compositions of single fibers from three muscles extracted from the same *Varanus salvator* specimen**.

**MHC**	**Biceps brachii No. of fibers**	**Caudofemoralis No. of fibers**	**Longissimus No. of fibers**
1	18	7	11
2	3	0	2
3	0	1	1
5	0	4	0
1, 2	2	3	7
2, 4	0	5	2
3, 5	0	3	0
5, 3	0	0	1

Note that at this level of analysis, these three muscles each showed unique patterns of MHC isoform distribution among individual fibers.

Mass spectrometry results provided substantial information for the identification of MHC isoforms expressed in the monitor heart and skeletal muscles. The three highest matches for each MHC band analyzed by mass spectrometry are listed in Table 4. A MOWSE score of ~70, with the mass tolerance set at 0.8 Dalton, is associated with a significant (greater than 95% probability) match. The MOWSE scores for the highest match for each band far exceeded this value, with a range of scores from 6609 to 9368.

**Table 4:** **Mass spectrometry results for the isoform identification of cardiac and skeletal muscle myosin heavy chain gel bands**.

**MHC Band**	**Match**	**Protein, Species**	**MOWSE Score**	**No. of Peptide Matches**
Atrial Band—slower migrating	Highest	Myosin-6, Mouse	6609	313
	Second	Myosin-6, Human	6319	302
	Third	Myosin-6, Rat	5890	289
Atrial Band—faster migrating	Highest	Myosin-7, Mouse	7995	365
	Second	Myosin-7, Bovine	7780	343
	Third	Myosin-7, Dog	7761	344
Ventricular band	Highest	Ventricular Myosin, Chicken	9368	397
	Second	Myosin-1, Dog	3030	127
	Third	Myosin-6, Human	2995	125
Skeletal band 1	Highest	Myosin-1B, Chicken	7209	332
	Second	Myosin-4, Dog	7042	300
	Third	Myosin, Adult skeletal, Chicken	6364	294
Skeletal band 2	Highest	Myosin-4, Dog	7870	312
	Second	Myosin-1B, Chicken	7417	319
	Third	Myosin-4, Pig	7101	282
Skeletal band 5	Highest	Myosin-7, Mouse	8367	935
	Second	Myosin-7, Dog	8040	907
	Third	Myosin-7, Horse	7868	898

The slower-migrating atrial MHC band matched with mammalian cardiac MHC-α. This is consistent with the results from the immunoblot with anti-MHC-α. The faster-migrating atrial band matched most strongly with mammalian cardiac MHC-β. This band, which co-migrated with band 5 in skeletal muscles, had the highest electrophoretic mobility of all of the MHC bands in monitor muscles and MHC-β in mammalian species virtually always is the fastest migrating band in samples containing cardiac and skeletal muscle sarcomeric MHC isoforms. Therefore, the two monitor atrial MHC isoforms appear to be orthologs of mammalian MHC-α and MHC-β. The strongest match of the single monitor ventricular band was with chicken ventricular MHC. This is consistent with the monitor MHC band never being observed in any other muscle and chicken ventricular MHC being restricted to only cardiac ventricle in adult chickens (Somi et al., 2006). Therefore, the monitor heart expresses orthologs of MHC isoforms that are expressed in mammalian and avian hearts.

Band 1 in monitor skeletal muscles matched most strongly with chicken MHC-1B, which is a fast-type isoform. The strongest match of monitor band 2 in skeletal muscle was with mammalian (dog) MHC-IIB, which is also a fast-type isoform. Bands 1 and 2 in monitor skeletal muscle were frequently found to be co-expressed in single fibers, so their identification as both being of the same type (fast) MHC isoforms is not surprising. Band 5 in monitor skeletal muscle, which always co-migrated with the fast-migrating MHC isoform in atrial muscle, matched most strongly with cardiac MHC-β. Slow-type MHC in mammalian striated muscle is referred to as MHC-β, in cardiac muscle, and MHC-I, in skeletal muscle. Therefore, monitor band 5 appears to be an ortholog of mammalian MHC-I/β. As stated above, monitor skeletal muscle band 4 was not identified by mass spectrometry, due to contamination from a closely-migrating band. This isoform co-migrated with the slower-migrating atrial isoform on one gel format, but they did not co-migrate on another gel format. Therefore, this band cannot be cardiac MHC-α, and the dot blot with anti-MHC-α indicated that MHC-α is not expressed in any of the examined monitor skeletal muscles. The identity of band 4 in skeletal muscle remains to be determined. However, band 4 was always co-expressed with band 2, which the mass spectrometry results indicate is a fast-type MHC isoform. Therefore, it is likely that band 4 is also a fast-type isoform or that the fibers which co-express bands 2 and 4 are hybrid fibers, co-expressing slow and fast MHC isoforms. Similarly, band 3, also not identified, was expressed either as the exclusive isoform in some fibers or was co-expressed with only band 5, which the mass spectrometry results indicate is slow-type MHC-I/β. Therefore, band 3 is likely to be a slow-type MHC isoform or, if it is a fast-type isoform, is occasionally expressed in other slow/fast hybrid fibers along with MHC-I/β. Unidentified bands 3 and 4 constituted only ~6–15% percent of the total MHC in the muscles in which MHC composition was quantitated (Table 2).

## Discussion

The results indicate that *V. salvator* iliocaudalis, longissimus, and caudofemoralis muscles differ markedly from each other at multiple levels. Presumably, these differences reflect muscle-specific roles during locomotion. In all three muscles, MHC was expressed most abundantly as bands 1 and 2. The two axial muscles (iliocaudalis and longissimus) had a similar distribution of myosin heavy chain isoforms, whereas the caudofemoralis differed by having greater amounts of bands 3 and 5. The MHC isoform profiles differed from what was seen at the enzymatic level where all three muscles were similar in having a majority of SO fibers (Table 1). The fast twitch index (the percentage of FG and FOG fibers) for these muscles was similar (caudofemoralis: 28%; iliocaudalis: 35%; longissimus: 39%) and much lower than those of the shoulder muscles of *Varanus exanthanthematicus*, which were all greater than 74% (Young et al., 1990). The oxidative index (the percentage of SO and FOG fibers) was lowest in longissimus (70%) and similar in caudofemoralis (89%) and iliocaudalis (90%); most of the appendicular muscles studied by Young et al. (1990) had oxidative indexes ranging from 55 to 70%.

At the level of whole muscle physiology, there was a consistency within the temporal data (Table 1). Caudofemoralis had both the slowest contraction speed and the lowest fatigue rate, while the appendicular muscles had faster contraction speeds and higher fatigue rates. The appendicular muscles studied by Young et al. (1990) had much shorter contraction times (approximately 70 ms) and did not exhibit a strong relationship between contraction time and fatigue index. The kinetics pattern was difficult to relate to the enzymatic fiber type profiles. Caudofemoralis and iliocaudalis had similar oxidative indexes, yet the former contracts approximately 34% slower and fatigues at half the rate of the latter (Table 1). A similar lack of clear relationship between fiber type profile and either contraction time or fatigue rate was found in the earlier study (Young et al., 1990). Variations in the relative cross-sectional area formed by the slow fibers could be present between muscles; such variation would not be reflected in the fiber type distribution but could result in variation in aspects of the whole muscle physiology.

The longissimus was the stiffest of the three muscles examined, and had an active force curve that closely paralleled the passive force curve (Figure 7). This was congruent with the EMG findings which indicate that this muscle was active during only one portion of the tail oscillation cycle (and thus one range of lengths) and the interpretation that this muscle is activated in a relatively steady-state manner. The iliocaudalis exhibited greater compliance in the passive force curve than did the longissimus; the active force curve for this muscle was more asymmetric (skewed toward elongation) and larger in proportion to the passive curve (Figure 7). This pattern was consistent with the EMG finding that this muscle was active through the majority of the tail oscillation cycle (including both changes in direction) and with the hypothesis that this muscle was one of the primary oscillators of the tail base. The compliance of the caudofemoralis was similar to that of the iliocaudalis, although the active force curve resembled, but was lower than, that of the longissimus (Figure 7). These findings were consistent with the hypothesis that this muscle could produce relatively low levels of force at any point of the tail oscillation cycle.

The EMG data suggests that, among the three studied muscles, the iliocaudalis was the primary oscillator of the tail; it was activated before tail displacement, showed a second burst during the change of direction, and scaled (both in onset time and duration of activity) with tail oscillation frequency (Figures 2–4). The longissimus (which was activated after the iliocaudalis) exhibited more of a steady-state activity pattern with no significant change in onset time or duration with increasing tail oscillation frequency (Figures 2–4). Differences in EMG activation pattern have been related to different basic swimming mechanics in fish (Knower et al., 1999). The tail of *V. salvator* accounts for nearly 50% of the total body length; if the longissimus exhibited the same “steady-state” pattern throughout the tail length, then disruption of the oscillation cycle would be likely at higher tail frequencies. Interestingly, our kinematic records frequently included disrupted oscillation cycles including “negative” oscillations in which the end of the tail was moving forward, or out of phase, with the rest of the tail.

When swimming, *V. salvator* retracts and adducts the hindlimbs. This posture shortens the caudofemoralis (Figure 1) and deprives this muscle of any real leverage with which to displace the tail. This was in sharp contrast to the situation in terrestrial locomotion in crocodilians in which the planted (and extended) hindlimb enables caudofemoralis to displace the tail (Gatesy, 1997). Herein an “opposite” function of caudofemoralis is proposed, namely that the muscle is acting on the femur to keep the hindlimb adducted against the tail base to minimize hydrodynamic drag (see D'Août and Aerts, 1997; Liu et al., 1997 for other examples of hydrodynamic hindlimbs). Our kinematic records show cyclic separation between the hindlimb and the tail (as the tail oscillates to the contralateral side of the body) which would be explained by the lag in the EMG onset for the caudofemoralis (Figure 2).

Despite the common name of “water monitor,” *V. salvator* is adept at climbing, digging, and cursorial locomotion (Burnell et al., 2012b). As such, care must be taken to not over-extend interpretation of muscle function based on analysis of only one locomotor mode (swimming). Still, active tail oscillation occurs only during swimming (Burnell et al., 2012b). The kinematic and EMG data strongly suggest that the appendicular muscle examined (caudofemoralis) does not function in oscillation of the tail, while the two axial muscles (iliocaudalis and longissimus) have different functions during tail oscillation.

The different functional roles proposed herein for the two studied axial muscles appear to be based on differences in muscle compliance and the activity patterns of the muscles (Figures 2, 7). These two muscles had similar enzymatic profiles (albeit with greater glycolytic activity in longissimus), whole muscle physiology, and MHC profiles (Figure 8, Tables 1, 2). Functionally, the caudofemoralis is herein interpreted as a true “appendicular” muscle functioning to adduct the hindlimb, rather than displace the tail. The caudofemoralis differed from the two axial muscles examined not only in the EMG patterns, but also in SO fiber content, slower twitch contraction time, greater force (both twitch and tetanic), highest compliance, and a different MHC profile. The properties of caudofemoralis differ significantly from those of the varanid appendicular muscles examined by Young et al. (1990) which were all predominantly fast twitch. It is not surprising to find discrepancies between EMG activity patterns and enzymatic fiber type profiles; in heterogeneous (non-compartmentalized) muscles like these, there is no way of knowing the enzymatic profile of the fibers we were recording the EMG signals from. But the difficulties in reconciling our different data sets go beyond this.

In both the present study of *V. salvator*, and the earlier study of *V. exanthamaticus*, there is little relationship between the enzymatic profile and the whole muscle physiological performance (in terms of both force production and fatigue). The relative lability of enzymatic fiber type categorization has long been recognized, particularly in relation to pH (e.g., Müntener, 1979). The categorization of the muscles examined in this study as predominantly Type I (slow twitch) fibers was supported by the slow contraction times recorded during the whole muscle physiology.

The analyses of muscle activation pattern, relative compliance, force production, MHC isoform composition, and fatigue were all able to distinguish among the three examined muscles. Categorization of the muscles, and differentiation between them, became more difficult at the biochemical and molecular levels. All three muscles had a similar preponderance of SO fibers (defined enzymatically), although the fast twitch and oxidative indexes could distinguish them. Similarly, the three muscles could be distinguished from each other on the basis of differences in MHC isoform expression. But it is important to note that the enzymatic and MHC isoform profiles, while both segregating caudofemoralis from the axial muscles, are not fully congruent in that the MHC isoform profiles suggest the muscles have a majority of “fast” myosin while the enzymatic results suggest a majority of “slow” fibers. The caudofemoralis was most distinct, differing from the other two with respect to the relative amounts of three MHC isoforms. Initial measurements (not shown) of shortening velocity in single fibers suggest that monitor fibers expressing bands 1 and/or 2 have markedly higher shortening velocity than fibers expressing the other MHC bands. The mass spectrometry results indicate that bands 1 and 2 are fast-type MHC isoforms. The caudofemoralis also had a greater amount of band 5 (i.e., MHCI/β), compared to the longissimus and iliocaudalis. These results provide additional insight into understanding the MHC isoform expression patterns among single fibers in the examined muscles and will guide interpretation of future measurements of contractile properties of single fibers in monitor muscles.

These findings suggest two, not mutually exclusive, explanations. First, the three muscles examined in the present study may truly be quite similar on a molecular and biochemical level, with the main functional differences observed arising out of higher-level specialization of the muscle tendon complex (compliance) and neural control (activation patterns). Second, the standard methodology for lizard enzymatic fiber typing (Gleeson et al., 1980) and the gel formats that were employed for MHC isoform analysis (although adequate to distinguish among the muscles) may not reflect the entire compositional diversity of these skeletal muscles.

In an attempt to explore the distinctions between our MHC isoform and enzymatic analyses, we sought to identify the observed MHC isoforms using not only mass spectrometry, but also their relationship to the cardiac MHC isoforms of *Varanus*, because it is well known that there is shared expression of some MHC isoforms in cardiac and skeletal muscles. Water monitor atrial and ventricular samples expressed different MHC isoforms. This is in contrast to mammalian species, which consistently exhibit two cardiac MHC isoforms, MHC-α and MHC-β (e.g., Hamilton and Ianuzzo, 1991). MHC-β is the same MHC isoform that is expressed in mammalian limb slow fibers, where it is referred to as MHC-I (e.g., Lompré et al., 1984; Yamauchi-Takihara et al., 1989; Rindt et al., 1993). The ventricular MHC and the predominant atrial MHC of the water monitor were identified as orthologs of chicken ventricular MHC and mammalian cardiac MHC-α, respectively (see Results). The monitor ventricular MHC was not observed to be expressed in any of the examined skeletal muscles and, given the presence of slow oxidative fibers in monitor skeletal muscles (this report), it appears that monitor slow fibers express an MHC isoform that is different from the ventricular MHC isoform. In fact, the mass spectrometry results indicate that monitor MHC band 5 is an ortholog of mammalian slow-type MHC-I/β and it is possible that this MHC is expressed in the histochemically-identified slow oxidative fibers. Changes in the MHC isoforms expressed in a given muscle can lead to distinct functional differences. Recent work has reported greater than predicted power output in larger lizards (James et al., 2015), and used an assumption of constant relative force production to look at scaling of limb mechanics in varanid lizards (Dick and Clemente, 2016); neither study addressed inter-specific variation in MHC isoforms, although such variation (if present) would clearly influence the conclusions in both papers.

It is possible that skeletal muscle fibers of *V. salvator* have characteristics that remain to be identified that support specific functions during species-specific locomotion. Earlier studies demonstrated that varanids have a specialized aerobic capacity compared to other lizards (reviewed by Frappell et al., 2002). Sustained activity levels in these lizards is supported by an efficient, and seemingly unique, unidirectional pulmonary airflow pattern (Schachner et al., 2014). High cardiac output is achieved by *Varanus* through specialized Ca^2+^ transport within the cardiac myocytes (Galli et al., 2009). Warren et al. (2010) also documented intracellular specializations within the cardiac myocytes of *Varanus*. It is possible that related intracellular specializations, such as glycogen stores, buffering systems, lactate transport capacity, the number and properties of mitochondria including the efficiency of oxidative phosphorylation, and enzymes that drive oxidative metabolism, occur within skeletal muscle fibers of *Varanus*. A more detailed analysis of the composition and functional performance of the skeletal muscle fibers may explain the unusual pattern of molecular, biochemical, and physiological properties that are reported in this study.

## Author contributions

BY was responsible for the overall project design; BY, JD, and MM performed the EMG experiments; BY, NJ, BL, AM, and MM performed the whole muscle physiology; NR and PR performed the molecular analyses of the muscle; BY and PR were primarily responsible for the drafting of the manuscript.

### Conflict of interest statement

The authors declare that the research was conducted in the absence of any commercial or financial relationships that could be construed as a potential conflict of interest. The reviewer HF and handling Editor declared their shared affiliation, and the handling Editor states that the process nevertheless met the standards of a fair and objective review.

## References

[B1] Ast J. C. (2001). Mitochondrial DNA evidence and evolution in Varanoidea (Squamata). Cladistics 17, 211–226. 10.1006/clad.2001.0169 34911248

[B2] Azizi E. Roberts T. J. (2014). Geared up to stretch: pennate muscle behavior during active lengthening. J. Exp. Biol. 217, 376–381. 10.1242/jeb.094383 24477610 PMC4008126

[B3] Bagnall M. McLean D. (2014). Modular organization of axial microcircuits in zebrafish. Science 343, 197–200. 10.1126/science.1245629 24408436 PMC4079086

[B4] Bergrin M. Bicer S. Lucas C. A. Reiser P. J. (2006). Three-dimensional compartmentalization of myosin heavy chain and light chain isoforms within dog thyroarytenoid muscle. Am. J. *Physiol. Cell Physiol.* 290, C1446–C1458. 10.1152/ajpcell.00323.2005 16371441

[B5] Bicer S. Reiser P. J. (2004). Myosin light chain 1 isoforms in slow fibers from global and orbital layers of canine rectus muscles. Invest. Ophthalmol. Vis. Sci. 45, 138–143. 10.1167/iovs.03-0716 14691165

[B6] Blough E. R. Rennie E. R. Zhang F. Reiser P. J. (1996). Enhanced electrophoretic separation and resolution of myosin heavy chains in avian and mammalian skeletal muscles. Anal. Biochem. 233, 31–35. 10.1006/abio.1996.0003 8789143

[B7] Bottinelli R. (2001). Functional heterogeneity of mammalian single muscle fibres: do myosin isoforms tell the whole story? Pflugers Arch. Eur. J. *Physiol*. 443, 6–17. 10.1007/s004240100700 11692261

[B8] Bredman J. J. Weijs W. A. Korfage H. A. Brugman P. Moorman A. F. (1992). Myosin heavy chain expression in rabbit masseter muscle during postnatal development. J. Anat. 180, 263–274. 1387129 PMC1259672

[B9] Bredman J. J. Wessels A. Weijs W. A. Korfage J. A. Soffers C. A. Moorman A. F. (1991). Demonstration of ‘cardiac-specific’ myosin heavy chain in masticatory muscles of human and rabbit. Histochem. J. 23, 160–170. 10.1007/BF01046587 1836206

[B10] Burnell A. Collins S. Young B. A. (2012a). The postpulmonary septum of *Varanus salvator* and its implication for Mosasaurian ventilation and physiology. Bull. Soc. Geol. France 183, 159–169. 10.2113/gssgfbull.183.2.159

[B11] Burnell A. Collins S. Young B. A. (2012b). Vertebral morphometrics in *Varanus*. Bull. Soc. Geol. France 183, 149–158. 10.2113/gssgfbull.183.2.151

[B12] Caldwell M. Sasso C. (2004). Soft-tissue preservation in a 95 million year old marine lizard: form, function, and aquatic adaptation. J. Vert. Paleo. 24, 980–985. 10.1671/0272-4634(2004)024[0980:SPIAMY]2.0.CO;2

[B13] Christian A. Garland T. (1996). Scaling of limb proportions in monitor lizards (Squamata: Varanidae). J. Herpetol. 30, 219–230. 10.2307/1565513

[B14] D'Août K. Aerts P. (1997). Kinematics and efficiency of steady swimming in adult axolotls (Ambystoma mexicanum). J Exp Biol. 200, 1863–1871. 9319776 10.1242/jeb.200.13.1863

[B15] Dick T. J. M. Clemente C. J. (2016). How to build your dragon: scaling of muscle architecture from the world's smallest to the world's largest monitor lizard. Front. Zool. 13:8. 10.1186/s12983-016-0141-5 26893606 PMC4758084

[B16] Fouré A. Comu C. McNair P. Nordez A. (2012). Gender differences in both active and passive parts of the plantar flexors series elastic component stiffness and geometrical parameters of the muscle-tendon complex. J. Orthop. Res. 30, 707–712. 10.1002/jor.21584 22034230

[B17] Frappell P. Schultz T. Christain K. (2002). The respiratory system in varanid lizards: determinants of O_2_ transfer. Comp. Biochem. Physiol. A 133, 239–258. 10.1016/S1095-6433(02)00147-2 12208298

[B18] Galli G. Warren D. Shiels H. (2009). Ca^2+^ cycling in cardiomyocetes from a high-performance reptile, the varanid lizard (*Varanus exanthematicus*). Am. J. Physiol. Reg. Integr. Physiol. 297, R1636–R1644. 10.1152/ajpregu.00381.2009 19812356 PMC2803631

[B19] Gatesy S. (1990). Caudofemoral musculature and the evolution of theropod locomotion. Paleobiology 16, 170–186. 10.1017/S0094837300009866

[B20] Gatesy S. (1997). An electromyographic analysis of hindlimb function in *Alligator* during terrestrial locomotion. J. Morphol. 234, 197–212. 10.1002/(SICI)1097-4687(199711)234:2<197::AID-JMOR6>3.0.CO;2-9 29852666

[B21] Gleeson T. T. Putman R. W. Bennett A. F. (1980). Histochemical, enzymatic, and contractile properties of skeletal muscle fibers in the lizard *Dipsosaurus dorsalis*. J. Exp. Zool. 214, 293–302. 10.1002/jez.1402140307 6456326

[B22] Hamilton N. Ianuzzo C. D. (1991). Contractile and calcium regulating capacities of myocardia of different sized mammals scale with resting heart rate. Mol. Cell. Biochem. 106, 133–141. 10.1007/BF00230179 1656210

[B23] James R. S. Vanhooydonck B. Tallis J. A. Herrel A. (2015). Larger lacertid lizard species produce higher than expected iliotibialis muscle power output: the evolution of muscle contractile mechanics with body size. J. Exp. Biol. 218, 3589–3595. 10.1242/jeb.124974 26417011

[B24] Kablar B. Krastel K. Ying C. Asakura A. Tapscott S. Rudnicki M. (1997). MyoD and Myf-5 differentially regulate the development of limb versus trunk skeletal muscle. Dev. 124, 4729–4738. 10.1242/dev.124.23.4729 9428409

[B25] Knower T. Shadwick R. Katz S. Graham J. Wardle C. (1999). Red muscle activation patterns in yellowfin (*Thunnus albacares*) and skipjack (*Katsuwonus pelamis*) tunas during steady swimming. J. Exp. Biol. 202, 2127–2138. 10409484 10.1242/jeb.202.16.2127

[B26] Lacquaniti F. Ivanenko Y. Zago M. (2012). Patterned control of human locomotion. J. Physiol. 590, 2189–2199. 10.1113/jphysiol.2011.215137 22411012 PMC3424743

[B27] Liu H. Wassersug R. Kawachi K. (1997). The three-dimensional hydrodynamics of tadpole locomotion. J Exp Biol. 200, 2807–2819. 9344964 10.1242/jeb.200.22.2807

[B28] Lompré A. M. Nadal-Ginard B. Mahdavi V. (1984). Expression of the cardiac ventricular alpha- and beta-myosin heavy chain genes is developmentally and hormonally regulated. J. Biol. Chem. 259, 6437–6446. 6327679

[B29] Müntener M. (1979). Variable pH dependence of the myosin-ATPase in different muscles of the rat. Histochemistry 62, 299–304. 10.1007/BF00508358 39907

[B30] Neyt C. Jagla K. Thisse C. Thisse B. Haines L. Currie P. (2000). Evolutionary origins of vertebrate appendicular muscle. Nature 408, 82–86. 10.1038/35040549 11081511

[B31] Osterlund C. Lindström M. Thornell L. E. Eriksson P. O. (2012). Remarkable heterogeneity in myosin heavy-chain composition of the human young masseter compared with young biceps brachii. Histochem. Cell Biol. 138, 669–682. 10.1007/s00418-012-0985-5 22777345

[B32] Pedrosa-Domellöf F. Eriksson P. O. Butler-Browne G. S. Thornell L. E. (1992). Expression of alpha-cardiac myosin heavy chain in mammalian skeletal muscle. Experientia 48, 491–494. 10.1007/BF01928171 1601115

[B33] Pianka E. King D. King R. (2004). Varanoid Lizards of the World. Bloomington, IN: Indiana University Press.

[B34] Rahnert J. A. Sokoloff A. J. Burkholder T. J. (2010). Sarcomeric myosin expression in the tongue body of humans, macaques and rats. Cells Tissues Organs 191, 431–442. 10.1159/000258678 19907142 PMC2859231

[B35] Rao M. Donoghue M. Merlie J. Sanes J. (1996). Distinct regulatory elements control muscle-specific, fiber-type-selective, and axially graded expression of a myosin light-chain gene in transgenic mice. Mol. Cell. Biol. 16, 3909–3922. 10.1128/MCB.16.7.3909 8668209 PMC231388

[B36] Reiser P. J. Bicer S. (2014). Electrophoretic separation of reptilian skeletal and cardiac muscle myosin heavy chain isoforms: dependence on gel format. Electrophoresis 35, 2615–2620. 10.1002/elps.201400221 24981405

[B37] Reiser P. J. Kline W. O. (1998). Electrophoretic separation and quantitation of cardiac myosin heavy chain isoforms in eight mammalian species. Am. J. Physiol. Heart Circ. Physiol. 274, H1048–H1053. 9530220 10.1152/ajpheart.1998.274.3.H1048

[B38] Rindt H. Gulick J. Knotts S. Neumann J. Robbins J. (1993). *In vivo* analysis of the murine beta-myosin heavy chain gene promoter. J. Biol. Chem. 268, 5332–5338. 8444907

[B39] Schachner E. Cleri R. Butler J. Famer C. (2014). Unidirectional pulmonary sirflow patterns in the savannah monitor lizard. Nature 506, 367–370. 10.1038/nature12871 24336209

[B40] Schilling N. (2011). Evolution of the axial system in craniates: morphology and function of the perivertebral musculature. Front. Zool. 8:4. 10.1186/1742-9994-8-4 21306656 PMC3041741

[B41] Somi S. Klein A. T. J. Houweling A. C. Ruijter J. M. Buffing A. A. M. Moorman A. F. M.. (2006). Atrial and ventricular myosin heavy-chain expression in the developing chicken heart: strengths and limitations of non-radioactive *in situ* hybridization. J. Histochem. Cytochem. 54, 649–664. 10.1369/jhc.5A6846.2006 16461363

[B42] Spangenburg E. E. Booth F. W. (2003). Molecular regulation of individual skeletal muscle fiber types. Acta Physiol. Scand. 178, 413–424. 10.1046/j.1365-201X.2003.01158.x 12864747

[B43] Vogel S. Labarbera M. (1978). Simple flow tanks for research and teaching. Bioscience 28, 638–643. 10.2307/1307394

[B44] Warren D. Galli G. Patrick S. Shiels H. (2010). The cellular force-frequency response in ventricular myocytes from the varanid lizard, *Varanus exanthemticus*. Amer. J. Physiol.: Reg. Integr. Physiol. 298, R567–R574. 10.1152/ajpregu.00650.2009 20053961 PMC2838664

[B45] Yamauchi-Takihara K. Sole M. J. Liew J. Ing D. Liew C. C. (1989). Characterization of human cardiac myosin heavy chain genes. Proc. Natl. Acad. Sci. U.S.A. 86, 3504–3508. 10.1073/pnas.86.10.3504 2726733 PMC287166

[B46] Young B. Boetig M. Fahey A. Lawrence A. (2008). The diversity of aquatic locomotion in extant varanoid lizards. Fort Hays Studies Spec. Iss. 3, 159–167.

[B47] Young B. Magon D. Goslow G. (1990). Length-tension and histochemical properties of select shoulder muscles of the savannah monitor lizard (*Varanus exanthematicus*): implications for function and evolution. J. Exp. Zool. 256, 63–74. 10.1002/jez.1402560109

